# Exploring the role of a participatory live music practice on nurses experienced levels of compassion satisfaction and fatigue: A mixed method study

**DOI:** 10.1371/journal.pone.0349801

**Published:** 2026-06-01

**Authors:** Nina M. van den Berg, Wolter Paans, Barbara van Leeuwen, Maya J. Schroevers, Hanneke van der Wal – Huisman

**Affiliations:** 1 Department of Surgery, University of Groningen, University Medical Center Groningen, Groningen, The Netherlands‌‌; 2 Research Group Nursing Diagnostics, Hanze University of Applied Sciences Groningen, Groningen, The Netherlands‌‌; 3 Department of Surgical Oncology, University of Groningen, University Medical Center Groningen, Groningen, The Netherlands; 4 Department of Health Sciences, section Health Psychology, University Medical Center Groningen, Groningen, The Netherlands; Federal University of Ceara, BRAZIL

## Abstract

**Objective:**

To explore nurses’ compassion satisfaction and compassion fatigue in relation to a participatory live music practice, and to understand nurses’ own perspectives on this dynamic.

**Introduction:**

A significant group of nurses experience compassion fatigue, the feelings of exhaustion and reduced capacity for empathy when caring for others, putting them at risk for work retention and turnover. Compassion satisfaction, the positive feelings derived from caregiving, may boost nurses’ well-being and resilience, and protects against compassion fatigue. Therefore, interventions enhancing compassion satisfaction provide dual benefits. This study examined to what extent an intervention based on participatory live music practice for nurses and their patients, by providing meaningful moments of compassion and bonding in the caring relationship, reduces nurses’ compassion fatigue and/or enhances their compassion satisfaction.

**Materials and methods:**

A mixed-method explanatory design was applied. First, exploratory quantitative data in 30 nurses were obtained via surveys prior to and after a week of participatory live music practice. Compassion satisfaction and compassion fatigue were assessed using the 21-item Professional Quality of Life Scale (ProQoL). Secondly, sequential semi-structured interviews with 22 nurses were carried out in two phases. Quantitative data was analyzed using descriptive statistics and Wilcoxon Signed-Rank Test. Qualitative data underwent thematic analysis.

**Results:**

Exploratory quantitative results suggested average levels of compassion satisfaction and fatigue, both before and after the week of participatory live music practice, with no significant changes over time. Qualitative results revealed two themes: (1) feelings of compassion satisfaction derived from observing patients’ response to the live music, leading to enhanced feelings of shared humanity and bonding, and a reconnection with nursing values; (2) perceived positive impact of the participatory live music on mood, work pleasure, and team dynamics.

**Conclusions:**

While quantitative data showed no significant changes in levels of compassion fatigue and satisfaction in the period before and after the live music practice, qualitative data revealed that nurses perceive benefits and compassion satisfaction by engaging in a participatory live music practice in the hospital setting.

## Introduction

Across cultures, live music fosters social bonding and enhances emotions like empathy and compassion [1]. In hospital settings, participatory live music practice can serve as a catalyst of compassionate contact between nurses and patients, supporting nurses in the delivery of compassionate care [2,3]. Compassion seeks to alleviate the suffering of others through relational understanding and action. It is coupled with empathy, the ability to understand and resonate with feelings of others [4].‌‌

In nursing, compassionate care involves recognizing a patients’ physical, emotional and psychological needs, coupled with the motivation to provide person-centered care that attributes to holistic relief [5]. It encompasses both *emphatic concern*, the component of empathy that causes the recipient to recognize and understand the suffering of others, and *compassion,* the motivation and commitment to provide relief of suffering through adequate care. Compassion and empathy are fundamental to nursing and ingrained in its core ethical values, committing nurses to the delivery of holistic, person-centered care that respects each patient’s dignity and individuality [6,7].

The unique emotional demands of the nursing profession, closely linked to the concepts of compassion satisfaction (CS) and compassion fatigue (CF), imply the potential benefits of incorporating participatory live music in hospital settings for nurses.

Compassion satisfaction refers to the feelings of reward, fulfillment and purpose derived from caring for others, particularly in difficult circumstances. Witnessing patient improvements in well-being or receiving appreciation for the care they provide gives nurses the emotional reward needed to maintain engagement and commitment to caring work over time [8,9]. Inherent qualities such as compassion and empathy align with the practice and core values of nursing, as well as with ideas of ‘good nursing’ [10,11].

Studies show that satisfaction from caring for others play a pivotal role in nursing students ‘ motivation [12,13].

Compassion fatigue, on the other hand, is the state of mental and physical exhaustion characterized by detachment, emotional withdrawal and a reduced capacity for empathy. Also referred to as ‘the cost of caring’, it arises from prolonged exposure to trauma and suffering in demanding and stressful circumstances, where caregivers often feel unable to improve patient outcomes or provide effective relief [14,15].

Nurses’ frequent patient interactions make them more prone to compassion fatigue than other medical staff [16]. With prevalence rates up to 52.55%, recent studies report that compassion fatigue affects over half of the nursing population [17,18]. Compassion fatigue negatively impact nurses’ viability by influencing social, emotional and cognitive aspects [19]. It is associated with increased anxiety and depression, higher rates of clinical errors, and with decreased performance, job satisfaction and quality of patient care [20–22]. It is a key factor behind the ongoing workforce crisis and the rising global dropout rates among nurses. Addressing compassion fatigue through workplace interventions is thus critical to support job engagement, promote nurse retention, and create sustainable healthcare environments for the future [23–25].

Interventions that increase nurses’ compassion satisfaction can have dual benefits. Higher compassion satisfaction is associated with higher resilience and the use of coping strategies, and with lower levels of secondary traumatic stress. It inversely relates with compassion fatigue and burnout, mitigates the effects of job stress, and leads to greater overall job satisfaction in nursing. By enhancing compassion satisfaction, the risk of developing compassion fatigue is reduced [26–29].

Participatory live music practice for nurses and patients in a hospital setting can provide meaningful moments that increase empathy, compassion, and bonding in the caring relationship. By being a direct catalyst of emotional expression, participatory live music enhances feelings of shared humanity and optimizes the potential for compassionate interaction [2,3]. The meaningful moments shared between nurses and patients can add depth and value to caring relationships and enhance moments of compassionate care [2,3,30]. A growing body of evidence points to further benefits of live music for nurses’ well-being, such as reduced stress, anxiety and burnout, enhanced relaxation and improved emotional resilience [31–34]. However, a knowledge gap remains on how such interventions may impact aspects that are directly related to nurses’ job engagement and job satisfaction, such as compassion satisfaction and compassion fatigue. Most research thus far has focused on the impact of music on patient outcomes rather than on the well-being of healthcare staff [35]. This study is the first to explore nurses’ experiences with a participatory live music practice in relation to perceived levels of compassion satisfaction and compassion fatigue.

### The study

Aim: To explore to what extent a participatory live music practice in the hospital ward was associated with, and perceived as, enhancing compassion satisfaction and reducing compassion fatigue in nurses.

Research hypothesis: It is hypothesized that nurses who participate in participatory live music practice will describe positive experiences related to enhanced compassion satisfaction and reduced compassion fatigue at work.

## Materials and methods

### Design

Compassion satisfaction and compassion fatigue are well known concepts within quantitative research and form the core elements of the Professional Quality of Life Scale (ProQOL), a tool used to measure the balance of positive and negative effects of caring work [36].

However, compassion is a multifaceted concept with different social, philosophical and religious denotations that is often differently understood and experienced by individuals. Moreover, live music is a personal experience that individuals can describe using a variety of words and concepts, necessitating additional qualitative approaches to obtain a detailed understanding of nurses’ experiences with the participatory live music and its perceived impact on feelings of compassion satisfaction and fatigue [37,38]

This study therefore adopted a mixed-method explanatory sequential design as delineated by Ivankova et al. (2016) [39]. This design encompasses two distinct phases: an initial quantitative phase succeeded by a qualitative phase. The rationale behind selecting this design to address our research inquiries was twofold. Firstly, through the employment of a quantitative methodology and the utilization of a standardized, widely recognized, and validated questionnaire, our intention was to scrutinize potential temporal variations in levels of compassion satisfaction and compassion fatigue pre- and post-engagement in participatory live music sessions to identify input for the subsequent qualitative inquiry. Given the exploratory nature of this study, statistical analyses of pre- and post-measurements were conducted in a descriptive and hypothesis-generating manner, aimed at identifying preliminary patterns and trends rather than drawing confirmatory conclusions.

**Fig 1:**
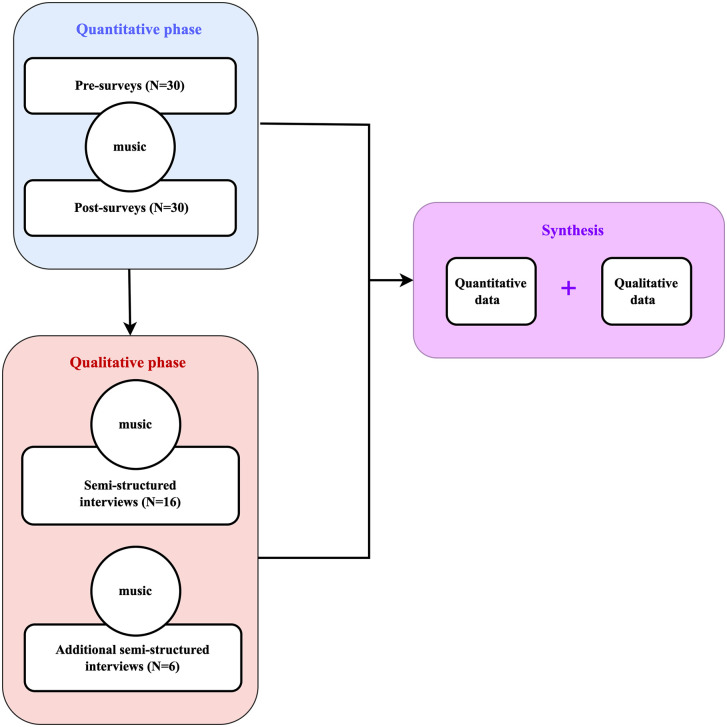
Research design.

Secondly, the qualitative inquiry via interviews was conducted to delve into nurses’ individual experiences concerning aspects associated with compassion satisfaction and fatigue, and how the participatory live music practice might have impacted these aspects. This qualitative component aimed to furnish deeper insights into the observed relationships identified during the quantitative phase. Fig 1 shows an overview of the process of data collection and data analysis.

### Research context

The perceived impact of a participatory live music practice among nurses and patients was studied in a research collaboration project between two Dutch hospitals and two conservatoires. A participatory live music practice is a form of participatory art engagement that requires little or no effort from its participants. Professionally trained musicians visit hospital wards and play for nurses and patients at the bedside. According to the musical preferences of the patient and the nurse, musicians play either from repertoire or create an improvisation on the spot [40]. From October 2020 to October 2022, multiple research settings, so called ‘live labs’ were created. In total, seven live labs were conducted across two hospitals. One hospital (Haaglanden Medisch Centrum) included one participating ward, while the other (Universitair Medisch Centrum Groningen) included four wards (i.e., in total five distinct wards). Two wards organized two live labs, all other wards only one. During each live lab at a specific ward, a team of three professional musicians visited the ward daily between 09:30 and 13:00 for 4–6 consecutive days. Each day, the musicians visited patient rooms at the ward. On average, 15 patient rooms were visited each day*.* The musicians were accompanied by a mediator who facilitated smooth communication between musicians, patients, and the personnel at the ward. Each morning, the musicians first played for nurses at a suitable moment that was sought in consultation with the ward and the head nurse, often right after the morning break. Musicians then visited the bedside of patients together with nurses, with each individual session lasting roughly between 10 and 15 minutes. Data collection took place between October 2020 and October 2022, alongside the planning and execution of the seven live labs. Data were collected during and shortly after each live lab, through observational field notes, interviews, and questionnaires.

### Quantitative phase: sample, measure, and analyses

The sample, recruited through convenience sampling, included nurses from five wards in two hospitals, who had attended the participatory live music sessions, as their participation in these sessions was essential to the study. Inclusion criteria required nurses to be on day shifts during the participatory live music intervention and to provide informed consent. Exclusion criteria included nightshift nurses and those unwilling to consent. Nurses were recruited based on their shifts and consent to participate. All eligible nurses were invited, resulting in 30 participants completing both surveys. No formal sample size calculation was performed. The sample size was pragmatically composed based on the number of eligible nurses present during the intervention period and the duration of the study. The aim of the statistical analysis was therefore not to draw confirmatory conclusions regarding intervention effects, but rather to use inferential statistical tests in an exploratory and descriptive, hypothesis-generating manner to identify indicative trends in compassion satisfaction and compassion fatigue to enrich our qualitative data interpretation.

Compassion satisfaction and compassion fatigue were measured using the validated 21-item. Version of the Professional Quality of Life Scale (ProQOL-5). This scale is widely used to assess levels of compassion satisfaction and compassion fatigue among caregiving professionals, and includes three subscales: compassion satisfaction, secondary traumatic stress, and burnout. The combination of the latter two subscales assesses compassion fatigue (Stamm, 2010) [36]. The ProQOL-21 is a revised version of the ProQOL-5 and was developed to improve the measurement accuracy. The 21-item version of the ProQOL-5 consists of: 10 items for compassion satisfaction, 5 items for burnout, and 6 items for secondary traumatic stress (these latter 11 items are combined into a measure of compassion fatigue). Each item is rated on a five-point Likert scale ranging from “never” (1) to “very often” (5). Total scores could therefore range from 10 to 50 for compassion satisfaction, and from 11 to 55 for compassion fatigue, with higher scores indicating higher levels of the respective constructs. Recommended cut-points for compassion satisfaction: a score of 21 for 25^th^ percentile, score of 26 for 50^th^ percentile, and a score of 30 for the 75^th^ percentile. For compassion fatigue, these cut-points are 16, 20, and 25 respectively. These cut-points refer to low, average and high levels. Previous validation of the revised 21-item version demonstrated high internal consistency and strong reliability across subscales [41]. In this study, we also found high internal consistency for both subscales, with the compassion satisfaction scale having a Cronbach alpha of 0.744, and the compassion fatigue scale a Cronbach alpha of 0.849. Participants completed the questionnaire in the week before and in the week following the live music visits to the ward. This interval was chosen due to practical constraints of the live lab setting, including the scheduling of live music sessions within a defined project period, nurses’ rotating shift availability, and the need to minimize participant burden and attrition in a high-intensity clinical environment. This design was primarily intended to capture relative short-term changes over time rather than sustained changes over time.

For the analyses, descriptive statistics and normal distribution tests were conducted to assess sample homogeneity. Changes over time in compassion fatigue and compassion satisfaction were examined using the non-parametric Wilcoxon Signed-Rank Test (given small sample, and skewed distribution of scores).

### Qualitative phase: sample, measure, and analyses

Interviews were audiotaped and conducted face-to-face by two of the authors who are experienced in conducting semi-structured interviews and trained in qualitative research methods (NvdB, MS). The researchers approached nurses in person, providing an information sheet outlining the study’s purpose, their role, and data handling procedures. All nurses that were approached consented to participate in research. Upon consent, a suitable time and location to conduct the interview was arranged in consultation with nursing management. No relationship was established between the researchers and participants prior to study commencement.

The first set of interviews (n = 16) were guided by a topic list based on a literature review and the expertise of the research team, and contained open-ended questions about nurses’ personal experiences with the participatory live music practice and it’s dynamic with markers of compassion satisfaction and compassion fatigue. Probing questions were asked to obtain a deeper understanding of nurses’ perspectives of this particular dynamic. Guided by the information emerging from preliminary analysis of the first interviews, the topic list guiding the second set of interviews (n = 6) was refined in line with an iterative qualitative approach. It included several follow-up questions specifically about compassion satisfaction and compassion fatigue and were formulated to align more closely with how nurses would consider these concepts in practice. This additional inquiry helped to deepen our understanding of how participants perceived these constructs in real-world contexts.

Thematic analysis was then employed to analyze the qualitative data, allowing for the identification of recurring themes and patterns related to nurses’ experiences. The thematic analysis followed the steps described by Nowell et al [42] and included:

- Familiarization with the data through repeated reading and reflective note-taking (NvdB)
- Generation of initial codes (NvdB)
- Searching for emerging themes (NvdB, WP, MS, HvdW)
- Reviewing and defining themes (NvdB, WP, MS, HvdW)
- Producing a detailed analysis for each theme and identifying examples (NvdB)

Familiarization with the data involved repeated reading of the transcripts and reflective memo writing. Initial coding was conducted by the first author, who systematically coded the full dataset. To enhance credibility, two members of the research team (MS, HvdW) independently double-coded a subset of transcripts. Coding differences were discussed until consensus was reached. The emerging coding framework was iteratively refined through discussion within the multidisciplinary research team, allowing researcher triangulation and critical reflection on alternative interpretation. Analytic memos were maintained throughout the process to document coding decisions and emerging interpretations.‌‌

Themes were developed through an iterative process of reviewing, comparing, and refining codes, followed by defining and naming themes. Atlas.ti® version 24 was used to manage and organize the qualitative data [43].

Data collection stopped once data saturation was achieved, defined as the point at which additional interviews no longer generated new codes or themes. Saturation was determined through analytic discussions within the research team.

### Ethical considerations

According to the Medical Ethics board of the Universitair Medisch Centrum Groningen (UMCG), this study did not fall within the scope of the Dutch law of Medical Research Involving Human Subjects and dispensation for further assessment was provided (reference number: 248807). Pseudonymization of the data allowed for the preservation of anonymity and confidentiality of participating nurses in both the qualitative and the quantitative research. Written informed consent was obtained from all participants. The principles of the Helsinki declaration were observed, and common ethical guidelines were followed in this study. Data were protected in accordance with the Dutch regulations in force.

## Results

**Fig 2:**
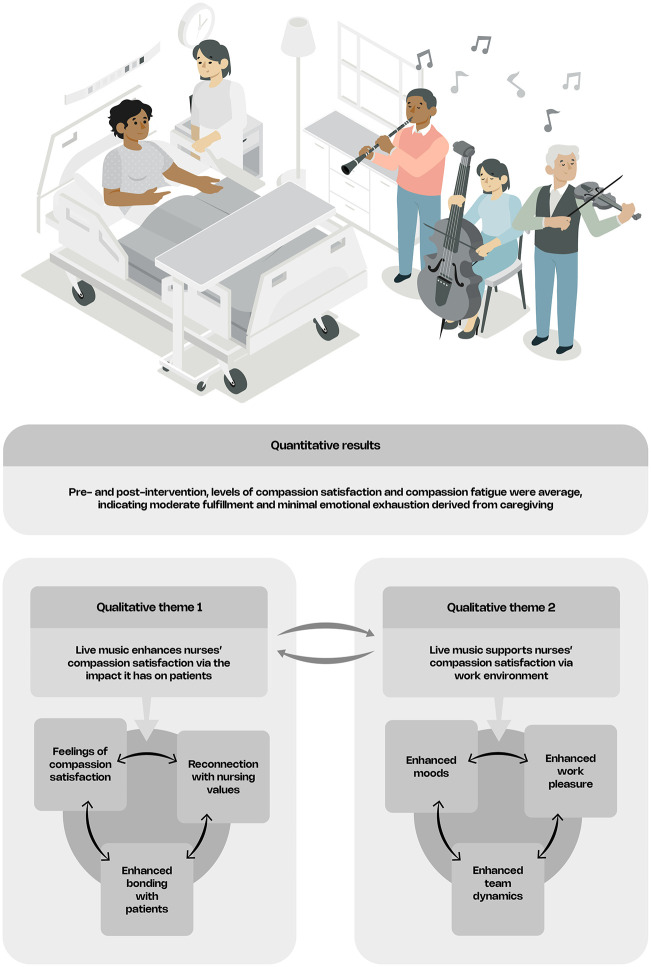
Interpretative model derived from thematic analysis. This figure presents an interpretative model derived from thematic analysis, illustrating perceived relationships between themes as described by participants, with quantitative findings presented descriptively.

An overview of both quantitative and qualitative results is depicted in Fig 2 and explained in more detail below.

### Quantitative results

Table 1. Median levels of CS and CF before and after the participatory live music practice.

**Table 1:** Median levels of CS and CF before and after the participatory live music practice.

	Pre intervention	Post intervention		
	Median	Median	p-value	Effect size (r)
Compassion Satisfaction	39.00	40.00	*0,209*	*0,23*
Compassion Fatigue	17.00	16.00	*0,542*	*0,11*

The Wilcoxon-rang test showed that nurses’ levels of compassion fatigue (Mdn_post_ = 16,00) and compassion satisfaction (Mdn_post_ = 40,00) after the participatory music sessions were not significantly different from their levels of compassion fatigue (Mdn_pre_ = 17,00 z = -,610, p = 0,542) and compassion satisfaction (Mdn_pre_ = 39,00, z = −1,256, p = 0,209) before the sessions. Thus, the research hypothesis, i.e., that nurses who participated in live music sessions will describe perceived increases in their compassion satisfaction and decreases in their compassion fatigue was not supported by the quantitative pre–post data. Given the recommended cut-points, nurses’ level of compassion satisfaction can be interpreted as relatively high both before and after the live music practice at the ward, and their level of compassion fatigue as relatively low.

### Qualitative results

Two main themes were derived from thematic analysis of the qualitative data, both relating to nurses’ perceived impact of the participatory live music in relation to aspects of compassion satisfaction. No findings concerning the participatory live music’s perceived impact on aspects of compassion fatigue were identified.

The first theme relates to what nurses experienced in response to their patients’ reaction to the live music sessions, and the second theme relates to what nurses directly experienced as a result of having participatory live music present at the ward. The identified subthemes under theme 1 mutually influence each other and exist in a cohesive rather than isolated manner, as depicted in Fig 2. The subthemes describe the different ways in which a participatory live music practice was experienced by care givers and care recipients by providing a meaningful moment that encouraged seeing each other beyond these roles, and how this was associated with aspects of compassion satisfaction.

### Theme 1: A participatory live music practice enhances nurses’ compassion satisfaction via the impact it has on patients

Throughout the interviews, nurses in this study prioritized the impact of the participatory live music practice on their patients as the primary outcome of the sessions. This initial focus shaped their perception of the overall added value of the practice for clinical environments and formed the foundation from which broader perceived benefits emerged. Three subthemes were identified, concerning how their patients’ reaction to the live music: (1) impacted nurses’ feelings of compassion satisfaction (2) allowed nurses to reconnect with their nursing values (3) enhanced bonding in the caring relationship.

#### Subtheme 1: Nurses derive feelings of compassion satisfaction from observing patient impact.

Nurses observed that the participatory live music practice both served as a catalyst for patients to express deep-seated emotions, ranging from joy to sadness, and as a means to reconnect with the outside world. It provided a distraction from the hospital environment and an opportunity for patients to re-identify with their humanity beyond their sickness or patient-status. Nurses witnessing this impact on patients reported feelings of fulfillment, gratitude and joy:

*“ I can hold onto such moments for days; it’s so touching. And it’s something truly special, just for them. I really believe it has an impact, especially when you’re in pain, helping you to relax and shift your focus.”* Nurse 16

For some nurses, these moments evoked emotional resonance, causing them to feel *with* their patients, and experiencing similar emotions as them during the participatory live music practice. This could improve bonding in the caring relationship (*see subtheme three*). Others strictly described how they felt *for* their patients, and how that impacted their personal feelings of satisfaction:

“*I think I just find it beautiful to see what it does to patients. I don’t necessarily feel emotional myself, but it does move me. And it also gives me a sense of fulfillment when you see that patients benefit from it, that it does them good.”* Nurse 7

The impact of the participatory live music practice on patients could also be experienced as contributing to nurses’ feelings of satisfaction by helping them achieve broader goals in providing care, such as ensuring patient comfort and well-being:

*“Someone happened to know he loved Bob Marley, which I didn’t know. It really made him light up. When a patient is happy and comfortable, I really enjoy that. His comfort is something you strive for in situations like this, so I think it definitely had impact on my job satisfaction”* Nurse 19

#### Subtheme 2: Observing patient impact allows to reconnect with nursing values.

Other nurses described how the patients’ reaction to the live music affirmed their role as confidante of the patient, clarifying the added value they have for patients in the hospital environment beyond their traditional role as provider of somatic care:

*We are a familiar and trusted presence for our patients, and they really appreciate having someone they know and trust with them during the music. And then, right after, they can say something to us like, ‘Oh, that was beautiful, wasn’t it? I really enjoyed that.’ The musicians come and go, but we stay, they have the opportunity to talk to us. I do think that adds value*” nurse 3

This allowed nurses to further reflect on their personal nursing values and reasons for choosing nursing as a profession, and reasserted their commitment to the social aspects of nursing:

*It kind of grounds you for a moment, like, yes, let’s just… just take a moment, because this is also what’s happening. It gives you a sort of realization, like, oh, we need to pay attention to this as well. I need to pay attention to this as well.* Nurse 21

It also confirmed their ideas about providing care in a person-centered way, and the importance of getting to know the patient ‘as a person’. Seeing how the participatory live music practice revealed their patients’ humanity helped them see new ways to optimize their relationships with patients:

“*I thought it was amazing. That man is not someone we know in that way, and we would never have come up with the idea ourselves to approach a conversation with him like that. And a lot of colleagues find it challenging too, as he is.. well, quite a grumpy man. So that makes it very special*” nurse 9

Witnessing how a moment of sincere personal attention boosted their patient’s morale also reminded nurses of the importance of caring for a patient’s needs in a holistic manner. It helped them to identify acts that could matter *to* the patient during hospitalization beyond medical or somatic care, and spoke with admiration of the perceived impact it had on patients’ behavior:

*“Especially room XX—he had been sleeping all morning, absolutely didn’t feel like doing anything. But the moment he heard that they had rehearsed a song just for him, that feeling of “they’re really here for me, to play a song” he sat up straight, started beaming all of a sudden. I hadn’t seen him like that yet. He also hadn’t been outside all day, but he just went outside for a walk. He was suddenly completely alive. I found that really beautiful to see, that music can do that.”* Nurse 19

#### Subtheme 3: Observing patient impact enhances bonding in the caring relationship.

Nurses described how participating in the live music practice together with patients could create meaningful moments, facilitating feelings of connection beyond their nurse/patient roles. Experiencing similar emotions as patients, or both being moved at the same time by a particular piece, could create enhanced feelings of bonding in the caring relationship:

*“When music is played, it really moves me. There might be a few tears, you know. It really gets to me. And when the patient is also enjoying it, and open to it, something special happens. I truly feel a connection with the patient in that moment, even if I’m sitting at a distance.”* Nurse 4

Experiencing bonding could lead to more meaningful conversations with patients that further boosted feelings of shared humanity between them. Nurses described how the opportunity to engage in different conversations with patients attributed to more joy and ease in delivering care:

*“They created a song incorporating a bit of his background and mine. So we had a conversation about the past and where we both came from. I ended up having really nice contact with that patient throughout their stay because we shared this with each other. You get to know each other just a little better, making the interaction smoother during the stay.”* Nurse 6

### Theme 2: A participatory live music practice impacts nurses’ compassion satisfaction by enhancing their work environment

While nurses in this study did not prioritize the participatory live music practice’s direct impact on themselves as an outcome or goal of the sessions, they did speak of how having music at the ward positively impacted their work surroundings, allowing them to feel more at ease and enabling them to derive more joy from work. Three subthemes were identified, concerning how the participatory live music attributed to (1) enhanced moods (2) enhanced team dynamics (3) enhanced pleasure derived from work.

#### Subtheme 1: Participatory live music impacts nurses’ compassion satisfaction by attributing to enhanced moods.

Nurses described that hearing live music at the ward, either in the background or while present during the performances, added to positive feelings and enhanced moods for themselves and others. For example, they spoke of the live music’s relaxing effect, that allowed them to pause momentarily and regain a sense calm in hectic environments:

“*I think everyone becomes a bit calmer, a bit more relaxed because of it. And more at ease—it puts things into perspective, I think. When you feel like you’re so busy, and you see what it does for the patients, it also reflects a little on yourself. You hear the music too, you enjoy it, and then… you feel ready to carry on, so to speak.”* Nurse 5

Having live music present was also described as uplifting, bringing joyful feelings and a change of scenery that positively impacted nurses, their colleagues and the broader healthcare community at the ward:

*“I notice that the atmosphere is different when you hear music around you for a few hours. It really does something to you, for your mood—you just feel calmer. I can see that everyone feels a bit more relaxed, or enjoys it, even the doctors are like, ‘Wait, is there music?’ Everybody ends up liking it.”* Nurse 13

#### Subtheme 2: A participatory live music practice impacts nurses’ compassion satisfaction by enhancing team dynamics.

Nurses spoke of how the participatory live music practice allowed them to share meaningful moments with colleagues. Experiencing such moments together and witnessing how colleagues could also become fully immersed or moved by the live music, could strengthen a sense of unity among them:

“*Well, it actually brings an even deeper connection with each other. In this department we share both joys and sorrows, and we’re really committed to providing good care. That’s why we ask a lot of ourselves, and of each other. It’s wonderful to be able to share these moments together, that you can build on further together*.” Nurse 8.

Nurses also mentioned that their patients’ reaction to the live music was a topic of discussion among them, and described how having that point of reference allowed for a different type of communication with team members:

*“With music, you notice there’s space to discuss other things, like “Oh wait, have they already been to your patients?” or “Where you there, what did you see?” It really does something for the atmosphere at work, for the dynamic between colleagues.*” Nurse 5

#### Subtheme 3: A participatory live music practice impacts nurses’ compassion satisfaction by stimulating work pleasure.

Having live music present at the ward was also perceived as attributing to the pleasure nurses derived from work. Nurses spoke of this as resulting from the participatory live music’s impact on emotions and feelings, both on patients, nurses, and the broader healthcare community, and how this created an open atmosphere in which compassion could flourish, attributing to enhanced social dynamics at the ward:

“*Yes, I work more comfortably. I’m someone who enjoys working with people, so that’s central to my role as a nurse. For me, the relationship with patients is far more important than the technical aspects of nursing. And when I experience that music with them, see that it has moved people, I feel happier. It makes my work more enjoyable*.” Nurse 4

Moreover, nurses described how it provided a welcome break of routine, and of their usual ways of thinking an acting in their professional roles. Most nurses mentioned ‘loving’ music, and experienced having music at the ward as means to connect with others in a meaningful way, making their workday less stagnant and allowing for new ways of bonding with one another:

*“You have a bit more interaction with everyone. Often, it’s hectic, with this and that going on. But by giving everyone a say and then coming to a shared outcome – which, in this case, was that song – it created a sense of togetherness. You’re all listening together to a song you’ve created as a group, that’s what made it lighter and more fun”* nurse 19.

## Mixed methods findings – integration of quantitative and qualitative results

In this section, we describe the combined quantitative and qualitative results to provide a comprehensive understanding of the benefits of a participatory live music practice on compassion satisfaction and fatigue among nurses. The exploratory quantitative data, using a validated scale to measure compassion satisfaction and compassion fatigue, suggest that nurses reported relatively high levels of compassion satisfaction and low levels of compassion fatigue, both before and after engaging in the live music practice. At the same time, in the qualitative interview findings conducted after having engaged in the live music practice, nurses reported several benefits from participating in the live music practices. As such, the qualitative data, obtained through in-depth interviews, uncovered personal and momentary experiences that were not observed when analyzing the quantitative data collected via a validated, standardized self-report scale. During the interviews, nurses reported: (1) perceived feelings of compassion satisfaction, in response to seeing how patients were affected by the live music, which allowed enhanced bonding in the caring relationship and a reconnection with nursing values; (2) a perceived positive influence of the participatory live music on their mood, work pleasure, and on team dynamics.

The discrepancies between the quantitative and qualitative findings may relate to differences in methodology as well as to measurement limitations (i.e., a standardized questionnaire such as the ProQol may overlook nuances), a Hawthorne effect and social desirability tendences in interviews, the limited exposure time to the live music sessions, and selection bias, as nurses with a pre-existing interest in music-based interventions or compassionate care may have been more likely to take part. Also, the novelty of the intervention may have temporarily enhanced participants’ perceived experiences, potentially contributing to more positive qualitative responses, that were not observed in the quantitative results.

## Discussion

In this study the perceived influence of participatory music practice on compassion satisfaction and compassion fatigue in nurses was explored. The mixed-method approach revealed a comprehensive understanding of the potential contribution of live music practice at the ward on nurses. Qualitative data revealed perceived benefits related to compassion satisfaction, such as more meaningful interactions between nurses, a sense of bonding, and personal and professional well-being. Yet the quantitative data did not show significant change in compassion satisfaction and fatigue, when measured by a validated, standardized measure. These rather stable levels may relate to the short study period and limited dosages of receiving the live music practice for nurses. The practical constrains of executing a participatory live music practice on nursing wards often allowed only short exposure in a dynamic environment, depending on nurses’ working schedule and clinical duties at the moments when the musicians were present at the ward. In addition, a generic measure of compassion satisfaction and fatigue such as the ProQOL, although validated, may lack the nuance and sensitivity to detect changes over time in this specific context. The short-term and situational effects of the intervention may not be adequately captured by an instrument primarily designed to assess more stable aspects of professional quality of life. The strength of the current study is that the ProQOL was complemented with a qualitative inquiry for a richer understanding.

The quantitative data provided an understanding of participating nurses’ average levels of professional quality of life in terms of their overall levels of perceived compassion satisfaction and compassion fatigue. Our findings indicated that the levels of compassion fatigue in this sample of nurses was relatively low, which contrasts with prior literature that identifies compassion fatigue as a significant issue. For instance, a recent systematic review and meta-analysis by Xie et al. (2021) found a pooled prevalence rate of 52.55% across international nursing samples [17]. Similarly, a qualitative study by Zhang et al. (2023) highlights how compassion fatigue is deeply embedded in nurses’ psychological experience of care delivery [18]. A possible explanation for the relatively low to average levels of compassion fatigue in our sample is that nurses who were willing to participate in this study may have been naturally more attuned to compassion satisfaction and compassionate care, and experienced relatively low levels of compassion fatigue.

To gain deeper insight, qualitative data collection complemented the ProQOL survey, allowing for an exploration of the transformative, interpersonal, and emotional aspects of nurses’ experiences with the participatory live music practice. These findings demonstrated that, despite short exposure to the intervention, the participatory live music practice was experienced as creating meaningful moments with patients. These moments were described as contributing to nurses’ compassion satisfaction and work pleasure and as humanizing their experience of the clinical environment.

Nurses described the participatory live music as a meaningful catalyst of emotions that created moments of sincere personal attention, allowing them to relate to their patients from a place of shared humanity rather than professional distance. This encouraged nurses to engage more deeply with the social aspects of their work, strengthening their caring relationships and reconnecting them with their core nursing values.

A novel concept emerged in this study: nurses described experiencing compassion satisfaction in relation to observing the live music’s perceived positive effect on their patients. This was associated with a strengthened sense of compassion and empathy and with more meaningful interactions with patients, colleagues, and the broader healthcare community at the ward. This element related to nurses’ compassion satisfaction was not captured by the standardized ProQOL survey, which measures general constructs rather than personal, momentary and context-specific experiences.

The results of this study contribute to the growing body of evidence on how the arts can positively influence healthcare environments, practices and outcomes.

Prior studies affirm that the shared emotional experience of participatory live music can humanize clinical environments by reducing stress, enhancing moods, and improving communication and bonding among participants [44,45]. By breaking barriers and fostering mutual empathy and understanding, it can also improve caregiving relationships between nurses and patients and support nurses in the provision of person-centered, holistic care [2,3].

The fact that even brief exposure to participatory live music practice was described as giving rise to meaningful experiences for both nurses and patients points to the untapped potential of these initiatives. Our findings suggest that it may relate to aspects of nurses professional quality of life. However, these findings were primarily reflected in the qualitative data and were not detected through the standardized quantitative measures.

Engaging in meaningful caregiving experiences, described in our findings in relation to participatory live music, creates a deeper sense of professional purpose that allows nurses to see their work as meaningful rather than transactional [46,47]. Additionally, it strengthens *compassion competence*, the ability to respond effectively to patients’ needs with sensitivity, intuitive insight, and adequate communication skills. A work environment that stimulates meaningful nursing and compassion competence strengthens nurses’ emotional resilience and work well-being, enabling them to experience greater compassion satisfaction, enhancing patient outcomes and significantly reducing burnout and compassion fatigue among nursing staff [4,48–50].

As an exploratory study, this research was intentionally small in scale and limited in duration to align with the practical constraints of the clinical setting. While the absence of a control group and the short pre-post design restrict causal inference, the combination of qualitative insights and validated measures offered valuable first indications of how live music interventions may affect nurses’ experiences. In this context, the statistical analysis aimed not to establish causality, but to explore whether preliminary signals of change could be detected that might inform the qualitative findings*.* Although the short pre-post interval aligned with the practical realities of the live lab context, it inherently limited the capacity of the design to detect changes in compassion satisfaction and fatigue. Given that the impact of live music interventions may be transient and context-dependent, longer follow-up periods would be necessary to determine sustained changes in compassion satisfaction and fatigue over time. Future research should consider larger samples, longer and repeated exposure to the intervention, and extended follow-up measurements (e.g., several weeks or months post-intervention), to examine both short- and long-term effects, assess the durability and potential consolidation of these effects, and clarify the mechanisms of impact.

Future research should also explore the long-term influence of participatory live music practice on nurses’ sustained professional quality of life and commitment to caregiving roles. The concise yet profound impact of these moments showcases the efficiency and scalability of integrating similar interventions into clinical settings, even when resources are limited, or target groups are constricted by time pressures.

While this study focused on the immediate impact of participatory live music, future investigations could examine its role in mitigating other challenges associated with task-centered care models, such as moral distress and emotional exhaustion.

This study also emphasizes the importance of a mixed method approach in evaluating context-dependent interventions even though mixed methods and qualitative research methods are not commonly applied in research in the medical sector. While quantitative tools such as the ProQOL scale are valuable, they alone could not capture the nuanced experiences and novel insights revealed through the qualitative approach. Future research should consider combining methodologies to comprehensively assess the impact of arts-based interventions in healthcare settings.

Investigating whether repeated or prolonged exposure to participatory live music enhances resilience and job satisfaction, reduces compassion fatigue, and bolsters retention in the nursing profession will be particularly valuable in this time of age, as modern clinical environments increasingly focus on output, efficiency and task-centered care [51–54]. This emphasis causes an inability to provide good nursing care -either according to ethical guidelines or nurses own standards- and creates the perception of continuously falling short, leading to guilt, moral distress, compassion fatigue, and a diminished sense of professional value, that causes major retention issues in nursing [55]. The continuous disappointments that nurses experience in regard to the emphasis on output rather than the quality of care, is also described as *psychological contract breach*: the discrepancy between expectations and values about nursing on the one hand, and the reality of the work on the other, that causes many nurses to ultimately leave the profession because it no longer aligns with their original vision and values [56–58].

In an era where the demands of clinical environments often risk eroding the humanistic aspects of caregiving, creating space for meaningful interactions in nursing is not a luxury but a necessity. Given the rising rates of burnout and attrition in the profession, humanizing healthcare environments and strengthening nurses’ sense of professional purpose is more critical now than ever [51,59,60].

This study underscores the potential of participatory live music to act as a catalyst for meaningful interactions related to nurses’ professional quality of life. By prioritizing empathy and humanity in care, the findings point to directions for future research into the longer-term role of art-based interventions in professional well-being and the broader caregiving experience. Additionally, this research highlights the value of applying mixed method or qualitative research methods in these contexts to adequately capture participants’ lived experiences and generate further insights into the emerging field of arts in health.

## Conclusion

Participatory live music practice was described as a catalyst of various emotions, with participants describing meaningful interactions between nurses and patients that stimulated a compassionate environment at the ward and a form of bonding beyond the conventional roles within clinical settings. The multiple positive experiences described in relation to these moments, both in terms of optimized nurse/patient relationships and its impact on the broader work environment, were associated with additional feelings of joy, satisfaction and meaning from work. This study highlights the potential for participatory live music practice to contribute to compassionate healthcare environments and the social aspects of nursing, with nurses describing additional meaning and fulfillment in their caring roles. Future research should explore the long-term role of participatory live music practices on both compassion satisfaction and fatigue, as well as their possible association with emotional distress and turnover intention among nurses.

## Supporting information

S1 File GRAMMS checklist.
Checklist for reporting qualitative and mixed-methods research.
(DOCX)

S2 File PLOS ONE Human Subjects Research Checklist.
Completed PLOS ONE checklist regarding human subjects research and ethics requirements.
(DOCX)
