# Supplementary material for: Exploring the role of a participatory live music practice on nurses experienced levels of compassion satisfaction and fatigue: A mixed method study
Source: PLoS One. 2026 Jun 1;21(6):e0349801. doi: 10.1371/journal.pone.0349801 (PMC13225627; doi:10.1371/journal.pone.0349801)
Supplement: S1 File — GRAMMS checklist. Checklist for reporting qualitative and mixed-methods research. (DOCX) [file pone.0349801.s001.docx]

**Appendix 1: Good reporting of a mixed-methods study (GRAMMS) checklist**

| **Guideline** | **Page information** |
| --- | --- |
| 1. Describe the justification for using a mixed methods approach to the research question | p. 5 |
| 2. Describe the design in terms of the purpose, priority and sequence of methods | p. 5 |
| 3. Describe each method in terms of sampling, data collection and analysis | p. 6 - 8 |
| 4. Describe where integration has occurred, how it has occurred and who has participated in it | p. 6 - 8 |
| 5. Describe any limitation of one method associated with the present of the other method | p. 16 - 17 |
| 6. Describe any insights gained from mixing or integrating methods | p. 17 – 20 |

**Reference:** O'Cathain A, Murphy E, Nicholl J. The quality of mixed methods studies in health services research. J Health Serv Res Policy. 2008;13: 92-98.
